# Reducing the time needed to administer a sustained attention test in patients with stroke

**DOI:** 10.1371/journal.pone.0192922

**Published:** 2018-03-22

**Authors:** Gong-Hong Lin, Ying-Pi Yang, Jeng-Feng Yang, Tzu-Ting Chen, Ching-Lin Hsieh

**Affiliations:** 1 School of Occupational Therapy, College of Medicine, National Taiwan University, Taipei, Taiwan; 2 Department of Pharmacy, Zhongxiao Branch, Taipei City Hospital, Taipei, Taiwan; 3 Department of Physical Therapy, College of Medicine, National Cheng Kung University, Tainan, Taiwan; 4 Department of Rehabilitation, Wanhua Hospital, Taipei, Taiwan; 5 Department of Physical Medicine and Rehabilitation, National Taiwan University Hospital, Taipei, Taiwan; 6 Department of Occupational Therapy, College of Medical and Health Science, Asia University, Taichung, Taiwan; University of Wuerzburg, GERMANY

## Abstract

Administering a sustained attention test often takes a lengthy time, which can hamper routine assessments in clinical settings. Therefore, we first proposed a method to reduce the time needed for administering a sustained attention test (the Computerized Digit Vigilance Test, C-DVT). The method was to retrieve 5 segments from different trial positions of the original C-DVT testing. Then we compared the concurrent validity, convergent validity, and random measurement error of the examinees’ performance on these segments to find the segment with better psychometric properties. The 5 segments were as follows: the first 50% of testing, the 21st~50th percentile of testing, the first 60% of testing, the 31st~60th percentile of testing, and the 36th~65th percentile of testing. Then we compared the validities and random measurement error of the examinees’ performance on these segments. Ninety patients with stroke participated in the validity study, and 44 of them participated in the random measurement error study. The patients’ scores on the 5 segments were highly correlated with those of the C-DVT (Pearson’s r ≥ 0.98), indicating excellent concurrent validity. The patients’ scores on the 5 segments were moderately correlated with those of the Tablet-based Symbol Digit Modalities Test (Pearson’s r = -0.51~-0.48), indicating sufficient convergent validity. The amounts of random measurement error (percent standard error of measurement) were all limited: 5.1% for the C-DVT, 6.6% for the first 50% of testing, 6.0% for the 21st~50th percentile of testing, 6.1% for the first 60% of testing, 6.0% for the 31st~60th percentile of testing, and 6.1% for the 36th~65th percentile of testing. The patients needed on average 3~4 minutes to complete all the aforementioned testing. The patients’ scores on the 5 segments showed excellent concurrent validity, sufficient convergent validity, and limited amounts of random measurement error in patients with stroke. We suggest the 31st~60th percentile of testing segment for users because it had the lowest amount of random measurement error and can reduce the time needed for formal testing by about 40%.

## Introduction

Sustained attention means the ability of a person to maintain a consistent behavioral performance on a task over a prolonged period of time [1]. Sustained attention is essential for mastering various daily tasks, such as driving, cooking, or working [2]. Patients with stroke often have deficits in sustained attention [2, 3]. Patients having deficits in sustained attention tend to have poor performance in postural control, mobility, and activities of daily living [2, 4]. Furthermore, such deficits may hamper patients’ motor and functional recovery[5] and are associated with increased risk of falls [2]. Thus, sustained attention is critical for patients with stroke receiving rehabilitation [6]. To manage issues of sustained attention for patients with stroke, a reliable (low random measurement error), valid, and fast-to-use measure assessing sustained attention is needed for clinicians.

However, administering a sustained attention test may take as long as 10 or more minutes (e.g., 14 minutes for the Conners’ Continuous Performance Test [7] and about 10 minutes for the Digit Vigilance Test, DVT) [8]. Such a lengthy test places great burdens on both clinicians and patients that can hamper the routine clinical assessment of sustained attention. A recent systematic review defines 3 minutes as the minimum time for testing sustained attention [6]. Thus, the time needed for administering measures of sustained attention leaves much room for improvement. A shortened measure would reduce assessment burdens and be welcomed by both clinicians and patients.

The Computerized Digit Vigilance Test (C-DVT) was designed for patients with stroke on the basis of the DVT to achieve a lower amount of random measurement error than that of the DVT [9]. The C-DVT was developed mainly by consulting with experts and field testing to minimize random measurement error. The results showed the C-DVT to have a limited amount of random measurement error [9]. The C-DVT is a computerized test, so it can easily be shortened by revision of the programming. To reduce the time needed for administering the C-DVT [9], we proposed 5 segments from different trial positions of the original C-DVT testing and compared the concurrent validity, convergent validity, and random measurement error of the segments with those of the original C-DVT in patients with stroke. If any proposed segments showed good validity and a limited amount of random measurement error, the segments of testing would lessen the burden of testing and increase the possibility of routine assessments of sustained attention in busy clinical settings.

## Materials and methods

### Subjects

We used the data from two of our studies. The first was an ongoing study examining the impact of attention on stroke recovery. The data were used for the validity investigation (i.e., concurrent validity and convergent validity) in this study. The second was a previous test-retest reliability study of the C-DVT [9]. The data were used for all the research purposes in this study. These two studies were approved by the Institutional Review Board of National Taiwan University Hospital (the reliability study of the C-DVT: 201303007RINC and the ongoing study: 201412187RINC). Each participant signed a written consent form.

In the ongoing attention study, the inpatients admitted to rehabilitation wards of a medical center were recruited consecutively, beginning in September 2014. The inclusion criteria were: (1) diagnosis of either ischemic stroke or cerebral hemorrhage, (2) onset of stroke within 3 months; (3) ability to follow 3-step oral instructions. We excluded patients with other neurological diseases (e.g., brain tumor or dementia) that may result in attention deficits. All the assessments were administered by a trained rater in a quiet room. The demographic and medical information was obtained from the patients’ medical records.

For the test-retest reliability study, the participants were recruited from the rehabilitation departments in three hospitals from July 2013 to February 2014 in a previous study [9]. The main inclusion criteria were: (1) diagnosis of either ischemic stroke or cerebral hemorrhage, (2) onset of stroke at least 6 months before the first assessment; (3) ability to follow 3-step oral instructions. We excluded patients with other neurological diseases (e.g., brain tumor or dementia) that could influence cognitive function. The participants were assessed twice, two weeks apart, by a trained rater in a quiet room. Further details were described in the previous study [9].

### Procedure

We first proposed a method of shortening the testing to reduce the time needed for formal testing of the C-DVT (120 trials). The method was to select 5 particular segments of the test from different trial positions. The time needed for formal testing of the C-DVT is about 6~10 minutes. We aimed to reduce that time by at least 1/3. We also retained about half of the original testing in order to meet the requirement of 3 minutes for a sustained attention test. Thus, the 5 segments of testing were retrieved as follows: the first 50% of testing [patients’ performance on the first 50% of the trials (i.e., the first 60 trials)], the 21st~50th percentile of testing, the first 60% of testing, the 31st~60th percentile of testing, and the 36th~65th percentile (middle 30%) of testing. That is, we retrieved the patients’ performance scores on these segments for further analysis. We expected that the time needed for the shortened method would be around 3~5 minutes. The session for examinees to practice the trial of the C-DVT (28 trials) was unchanged.

### Measures

The C-DVT was developed on the basis of the DVT [9]. There are 28 trials in a practice session and 120 trials in a formal test. The main devices of the C-DVT are a computer screen and an external keyboard with two buttons [a circle (“O”) and an X (“X”)]. When taking the C-DVT, examinees are required to judge whether the screen shows the digit “6” in a vertical column of 5 digits. The column is placed at the center of the screen, which is designed for patients who might have spatial neglect. If the screen shows the digit “6”, examinees should press the “O” button using the index finger of their dominant hand; otherwise, they should press the “X” button using the middle finger of their dominant hand. The response-stimulus interval and the inter-stimulus interval of the C-DVT are the same. The interval is 0.5, 1.0, or 1.5 s, which is randomly chosen. The time (in s) needed for completing each trial is automatically recorded. The number of errors is also recorded. The total time for completing the C-DVT reflects examinees’ ability of sustained attention. A shorter time indicates better ability of sustained attention. The C-DVT has good test-retest reliability, concurrent validity, and ecological validity in patients with stroke [9] and schizophrenia [10].

The Tablet-based Symbol Digit Modalities Test (T-SDMT) was designed on the basis of the SDMT [11]. The T-SDMT includes 9 symbols (e.g., □, Σ, and Δ), each corresponding to an Arabic numeral (1–9), presented to the examinee on the iPad screen. To respond to each stimulus, the examinee is required to look at the stimulus (a symbol) in the center of the screen, then search for the corresponding number of the stimulus in the table at the top of the screen, and finally choose the corresponding number on a 3*3 grid at the bottom of the screen. The iPad automatically records the number of correct answers during 90 seconds of the formal testing. A higher number of correct answers reflects better performance of information processing speed. The T-SDMT has sufficient test-retest reliability and concurrent validity in patients with stroke [11].

The Barthel index (BI) is a measure of disability [12]. Wade and Collin’s version was used [12]. Its score ranges from 0 to 20 (higher scores indicating less disability). The BI has sufficient reliability and validity in patients with stroke [13, 14].

### Data analysis

Descriptive statistics were used to present the characteristics of the participants. To examine the concurrent validity of the proposed segments, we used the Pearson correlation coefficient (Pearson’s r) to examine the association between the scores obtained from the segments and those of the C-DVT. We expected that the level of association should be high in order to indicate good concurrent validity [15]. Thus, Pearson’s r > 0.75 was considered to indicate good concurrent validity of the proposed segments [16].

To investigate the convergent validity, we also used Pearson’s r to examine the association between the scores obtained from the segments and those of the T-SDMT (assessing information processing speed). Patients’ ability of sustained attention is substantially correlated with that of information processing speed. Their correlation cannot be very high because sustained attention and information processing speed are different *per se*. Thus, we expected that the level of association would be moderate in order to indicate good convergent validity. An absolute value of Pearson’s r around 0.50 was considered to indicate good convergent validity of the proposed segments [17].

To determine the amount of random measurement error of the patients’ performance on the 5 segments, we calculated the standard error of measurement (SEM) [18]. The SEM is an indicator of the degree to which measured test scores are spread around a “true” score [18–21], and thus it is an index of reliability [20]. The SEM is commonly estimated from the test-retest reliability of a test (Pearson’s r or intraclass correlation coefficient, ICC) [18]. The SEM percentage (SEM%, SEM divided by the mean of all test-retest scores) was used to compare the amount of random measurement error. An SEM% < 10% was considered to indicate a limited amount of random measurement error [22]. In addition, we calculated cumulative reliability (using SEM% as the reliability index in this study) over trials [23, 24].

We also calculated the minimal detectable change (MDC) [25], which means the minimal amount of change needed to determine whether a change score is beyond random measurement error at a certain level of confidence [18]. We used the 95% confidence level for the MDC. The MDC was calculated on the basis of the SEM.

For ease of comparison of SEM/MDC among the 5 segments, the scores obtained from the 5 segments were linearly transformed to be the same as those of the C-DVT. For example, we multiplied the scores obtained from the first 50% testing segment by 2.

## Results

Ninety patients participated in the validity study. Forty-four patients came from the previous test-retest study [9], and 46 came from the ongoing study examining the impact of attention on stroke recovery. The 44 patients were at the chronic stage after stroke, and the 46 patients were at the subacute stage, receiving hospital rehabilitation. On average, the 90 patients had mild to moderate disability according to their scores on the BI. Further details of the patients are shown in Table 1.

**Table 1:** Demographic characteristics and stroke-related information of the patients.

Characteristic	A previous test-retest study used for calculating SEM (n = 44)	An ongoing study (n = 46)	Our validity study* (n = 90)
**Gender**, n (%)			
Male	28 (63.6%)	35 (76.1%)	63 (70.0%)
**Age**, years, mean±SD	56.9±12.9	56.9±12.4	57.1±12.6
**Stroke type**, n (%)			
Cerebral hemorrhage	18 (40.9%)	12 (26.1%)	30 (33.3%)
Cerebral infarction	26 (59.1%)	34 (73.9%)	60 (66.7%)
**Side of hemiplegia**, n (%)			
Left	23 (52.3%)	32 (69.6%)	55 (61.1%)
Right	21 (47.7%)	13 (28.3%)	34 (37.7%)
Bilateral	0 (0.0%)	1 (2.1%)	1 (1.2%)
**Time since stroke onset**, months, median (1^st^~3^rd^ quartile)	21.2 (12.5~49.3)	0.9 (0.7~1.4)	2.3 (0.9~17.6)
**Barthel Index**, mean±SD	17.3±2.9	13.6±3.6	15.4±3.8
C-DVT			
Number of errors, median (1^st^~3^rd^ quartile)			
1^st^ assessment	1 (0~2)	3 (1~5)	2 (0~3)
2^nd^ assessment	1 (0~2)	-	-
Completion time, second, mean±SD			
1^st^ assessment	306.6±61.0	412.1±197.5	360.5±156.7
2^nd^ assessment	297.7±51.5	-	-

C-DVT: Computerized Digit Vigilance Test; SEM: standard error of measurement

*Combining the 1^st^ assessment data of the previous test-retest study and the data of the ongoing study.

The 90 patients took, on average, 360.5 seconds or 6.0 minutes (SD = 156.7 seconds) to complete the formal testing of the C-DVT. The completion time of the 5 segments was calculated from the patients taking the test in the corresponding session. However, the time needed for administering the 31st~60th percentile testing was the same as that for the first 60% of testing because the testing ended at the same trial. The mean time needed for completing any one of the 5 segments was estimated to be about 3~4 minutes (S1 Appendix).

Table 2 shows the results of validity investigations. For concurrent validity investigation, the scores of the 5 segments were highly correlated with those of the C-DVT (Pearson’s r ≥ 0.98). For convergent validity investigation, the scores of the 5 segments were moderately correlated with those of the T-SDMT (Pearson’s r ranging from -0.51 to -0.48). The patients’ scores on the C-DVT were also moderately correlated with those of the T-SDMT (Pearson’s r = -0.51).

**Table 2:** Concurrent validity and convergent validity of the 5 segments of the C-DVT (Pearson’s r, n = 90).

Measure	First 50% of testing	21st~50th percentile of testing	First 60% of testing	31st~60th percentile of testing	36th~65th percentile of testing	C-DVT
C-DVT	0.98	0.98	0.99	0.99	0.99	-
T-SDMT	-0.49	-0.48	-0.49	-0.49	-0.48	-0.51

C-DVT: Computerized Digit Vigilance Test; T-SDMT: Tablet-based Symbol Digit Modalities Test

**Fig 1:**
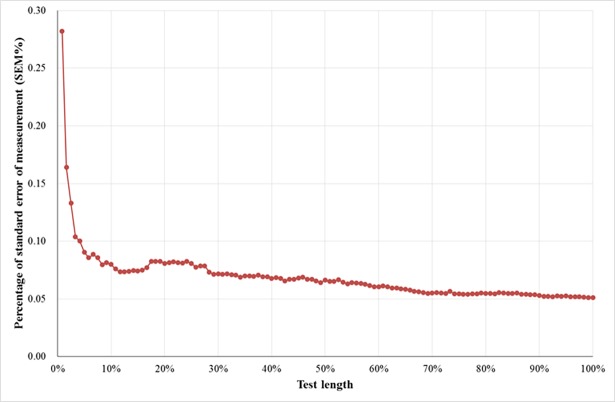
The cumulative reliability (SEM% calculated on the basis of ICC) over trials. The SEM% of each dot is the cumulative reliability (calculated from the first trial to that trial).

Table 3 shows the amounts of random measurement error of the 5 segments as compared to that of the C-DVT. The SEM% of the 5 segments ranged from 6.0% (the 21st~50th percentile of testing and the 31st~60th percentile of testing) to 6.6% (the first 50% of testing) and 5.1% for the C-DVT. Fig 1 shows that patients’ performance on the first 10% of testing was very unstable. After the first 10% or 30% of testing, the cumulative reliability (SEM%) was very stable. The above SEM (SEM%) was calculated on the basis of ICC. The SEM (SEM%) was also calculated based on Pearson’s r, and the results were very similar (S2 and S3 Appendices). ICC accounts for systematic bias (e.g., practice effect), and the corresponding values of SEM (SEM%) might have resulted in slightly larger values than those of the calculation based on Pearson’s r. Because the SEM (SEM%) calculated on the basis of ICC was more conservative, we adopted ICC and reported the resulting SEM (SEM%) in the main text.

**Table 3:** Standard error of measurement (SEM) and minimal detectable change (MDC) of the 5 segments and the C-DVT (n = 44).

Shortened method/ Measure	SEM* (seconds)	SEM%	MDC* (seconds)
First 50% of testing	20.5	6.6	56.7
21st~50th percentile of testing	18.5	6.0	51.4
First 60% of testing	18.7	6.1	51.9
31st~60th percentile of testing	18.5	6.0	51.2
35th~64th percentile of testing	18.7	6.1	51.8
C-DVT	15.7	5.1	43.5

*The scores used for calculating the SEM and MDC of the 5 segments were linearly transformed to be the same as those of the C-DVT. The SEM and MDC were calculated on the basis of ICC.

Table 3 also shows the MDC values [51.2 seconds (the first 60% of testing) to 56.7 seconds (the 31st~60th percentile of testing)] of the 5 segments for prospective users to determine whether the change score of an individual patient would be beyond random measurement error at the 95% confidence level. The MDC values of the 5 segments were linearly transformed for comparison with the C-DVT. In addition, the data used in this study are shown in S4 Appendix.

## Discussion

We found that the scores of the 5 segments were highly associated with those of the original C-DVT (Pearson’s r ≥ 0.98). The results mean that no matter which of the 5 testing segments was used, the resulting scores were almost the same as those of the original C-DVT. However, the results might have been overestimated because we obtained the scores of the 5 segments from the testing of the original C-DVT. Even though the extremely high association (Pearson’s r ≥ 0.98) was compromised, the true level of association between them would still be very high. These observations indicate that the 5 segments had excellent concurrent validity. The results also indicate that the patients’ sustained attention was generally consistent during the first two thirds of the testing (the testing periods of the 5 segments).

The scores obtained from the 5 segments were moderately correlated with those of the T-SDMT (Pearson’s r ranging from -0.51 to -0.48). These findings support our hypothesis that sustained attention would be substantially associated with the information processing speed of the participants. It is noted that the extent of association between these measures might have been affected by the level of reliability (or the amount of random measurement error) [21]. Fortunately, the reliability of these measures appeared satisfactory and similar to that found in this and previous studies [9, 11]. Thus, the extent of association between these measures might not be substantially compromised. In addition, the level of association was very close to that between the scores of the C-DVT and the T-SDMT (Pearson’s r = -0.51). These results indicate that the scores obtained from the 5 segments had sufficient convergent validity. These results further support the validity of the patients’ scores on the 5 segments in patients with stroke.

The results showed that the SEM% of the 5 segments was low (ranging from 6.0%~6.6%). Among the segments, the SEM% of the 31st~60th percentile of testing (6.0%) was close to that of the C-DVT (5.1%). These results indicate that the amount of random measurement error of the 5 segments was limited and satisfactory. These findings also indicate that the scores obtained from the 5 segments can be very reliable. It is noted that the SEM% of the 31st~60th percentile of testing (6.0%) was slightly smaller than that of the first 60% of testing (6.6%). Such a trend was also found for the 21st~50th percentile of testing (6.0%) and the first 50% of the testing (6.1%). Fig 1 shows that patients’ performance on the early trials was unreliable. After the first 30% of testing, the cumulative reliability (SEM%) was very reliable. These observations indicate that the patients’ responses were more stable in the middle and late stages of the testing and that the late stage of the testing may have been unnecessary.

The aforementioned findings and implications suggest that prospective users can use one of the 5 segments in both clinical or research settings. Each segment can serve as an alternative of the C-DVT and reduce the time needed for formal testing by about one third to one half. We suggest the 31st~60th percentile testing method to prospective users because it had the lowest amount of random measurement error among the 5 segments and reduced the time needed in formal testing by 40%. In addition, the mean time needed for completing the 31st~60th percentile testing segment was estimated to be about 3.5 minutes, which satisfies the requirement that sustained attention should be tested for at least 3 minutes [6]. The C-DVT is a computerized test, so the above testing methods can be installed on a mobile device. These features justify the feasibility of the shortened method.

The main purposes of our study were to shorten the C-DVT in order to lessen the burden of testing while maintaining sufficient psychometric properties. A lengthy test usually provides reliable results. However, a lengthy test may cause fatigue in examinees (particularly for patients with stroke), which would lead to fluctuations in the results. Some researchers have introduced methods (e.g., “occasional reminders to try-harder” [26] and “taking a rest” [27]) to avoid performance fluctuation and found positive results [26, 27]. Fortunately, our results showed positive findings for the proposed method. These observations support the notion that empirical evidence is critical for justifying any proposed methods to improve the utility while maintaining the quality of a clinical measure.

The scores obtained from the proposed segments can be linearly transformed to be the same as those of the C-DVT for comparison. For example, we can multiply the scores obtained from the first 50% of testing method by 2 and compare the transformed scores with those of the C-DVT. Such a transformation makes possible the interchangeable use of the 5 proposed segments and the C-DVT and further promotes the utility of the proposed segments. However, only a few errors (median ≤ 2) were made by the patients. The transformation may over- or underestimate the number of errors. Thus, the transformation may not be appropriate for errors committed.

The MDC of the 5 segments ranged from 51.2 to 56.7 seconds for the transformed scores. Clinicians can use the value of MDC as a threshold to determine whether the change score of a patient is due to random measurement error of the assessments. Researchers can employ the MDC to examine the effect of an intervention on every single patient [18, 28]. If a treatment effect (change score of a patient) cannot exceed the MDC (random measurement error), clinical users will be unable to find a true change (beyond random measurement error) of the patient [18, 28]. Thus, the MDC of the patients’ scores on the 5 segments is useful for both clinicians and researchers.

There are 3 limitations in this study. First, the data used in this study were retrieved from two studies (one study is ongoing and all the data are available on the journal’s web site) [9]. Our proposed method has not been used independently. Thus, our results might have been overestimated. Second, our proposed method was validated with the original C-DVT, not with other tests of sustained attention. Further studies should employ the methods independently and other well-known sustained attention tests (e.g., the d2 test of attention [29] or the Conners’ Continuous Performance Test [7]) to further confirm our findings. Third, examinees’ performances on the 5 segments of C-DVT need to be validated in healthy populations. A norm of the best shortened method is also needed. These studies would strengthen the utility of the shortened method.

## Conclusions

The patients’ performance on the 5 segments showed excellent concurrent validity, sufficient convergent validity, and limited amounts of random measurement error in patients with stroke. We suggest the 31st~60th percentile testing segment for prospective users because it had the least amount of random measurement error among the 5 segments and can reduce the time needed in formal testing of the C-DVT by about 40%.

## Supporting information

S1 Appendix Estimated completion time and number of errors of the 5 proposed segments. (DOC)

S2 Appendix Standard error of measurement (SEM) and minimal detectable change (MDC) of the 5 segments and the C-DVT (n = 44). (DOCX)

S3 Appendix The cumulative reliability (SEM% calculated on the basis of Pearson’s r and ICC, respectively) over trials. The SEM% of each dot is the cumulative reliability (calculated from the first trial to that trial).
(TIF)

S4 Appendix Raw data of this study. (XLSX)
