# Supplementary material for: Reducing the time needed to administer a sustained attention test in patients with stroke
Source: PLoS One. 2018 Mar 22;13(3):e0192922. doi: 10.1371/journal.pone.0192922 (PMC5863955; doi:10.1371/journal.pone.0192922)
Supplement: S1 Appendix — (DOC) [file pone.0192922.s001.doc]

**S1 Appendix. Estimated completion time and number of errors of the 5 shortened methods.*****

|  | A previous reliability study used for calculating SEM (n=44) | An ongoing study  (n=46) | Our validity study^*^  (n=90) |
| --- | --- | --- | --- |
|  | mean±SD or median (1^st^~3^rd^ quartile) | | |
| First 50% of testing |  |  |  |
| Completion time, second | 154.4±31.6 | 180.9±49.1 | 167.9±43.5 |
| Number of errors | 0.5 (0~1) | 1 (0~2) | 1 (0~2) |
| 21st~50th percentile of testing |  |  |  |
| Completion time, second | 154.4±31.6 | 180.9±49.1 | 167.9±43.5 |
| Number of errors | 0.5 (0~1) | 1 (0~2) | 1 (0~2) |
| First 60% of testing |  |  |  |
| Completion time, second | 184.5±37.1 | 217.3±59.3 | 201.3±52.3 |
| Number of errors | 1 (0~1) | 2 (0~3) | 1 (0~2) |
| 31st~60th percentile of testing |  |  |  |
| Completion time, second | 184.5±37.1 | 217.3±59.3 | 201.3±52.3 |
| Number of errors | 1 (0~1) | 2 (0~3) | 1 (0~2) |
| 35th~65th percentile of testing |  |  |  |
| Completion time, second | 200±40.6 | 235.7±64.9 | 218.2±57.2 |
| Number of errors | 1 (0~1.3) | 2 (0~3) | 1 (0~2) |

*The completion time and number of errors of the shortened methods were calculated from the patients taking the test in the corresponding segment.
