# Supplementary material for: Reducing the time needed to administer a sustained attention test in patients with stroke
Source: PLoS One. 2018 Mar 22;13(3):e0192922. doi: 10.1371/journal.pone.0192922 (PMC5863955; doi:10.1371/journal.pone.0192922)
Supplement: S2 Appendix — (DOCX) [file pone.0192922.s002.docx]

**S2 Appendix. Standard error of measurement (SEM) and minimal detectable change (MDC) of the 5 segments and the C-DVT (n = 44).**

| Shortened method/  Measure | SEM^*^  (seconds) | SEM% | MDC^*^  (seconds) |
| --- | --- | --- | --- |
| First 50% of testing | **18.9**^†^  20.5 | **6.1**  6.6 | **52.4**  56.7 |
| 21st~50th percentile of testing | **17.5**  18.5 | **5.7**  6.0 | **48.4**  51.4 |
| First 60% of testing | **16.8**  18.7 | **5.5**  6.1 | **46.7**  51.9 |
| 31st~60th percentile of testing | **16.2**  18.5 | **5.3**  6.0 | **44.8**  51.2 |
| 36th~65th percentile of testing | **15.1**  18.7 | **4.9**  6.1 | **41.9**  51.8 |
| C-DVT | **12.7**  15.7 | **4.1**  5.1 | **35.2**  43.5 |

^*^The scores used for calculating the SEM and MDC of the 5 segments were linearly transformed to be the same as those of the C-DVT.

**^†^**Values in bold type were calculated on the basis of Pearson’s r, and the rest were calculated on the basis of ICC.
