# Supplementary figures and images for: Reducing the time needed to administer a sustained attention test in patients with stroke
Source: PLoS One. 2018 Mar 22;13(3):e0192922. doi: 10.1371/journal.pone.0192922 (PMC5863955; doi:10.1371/journal.pone.0192922)

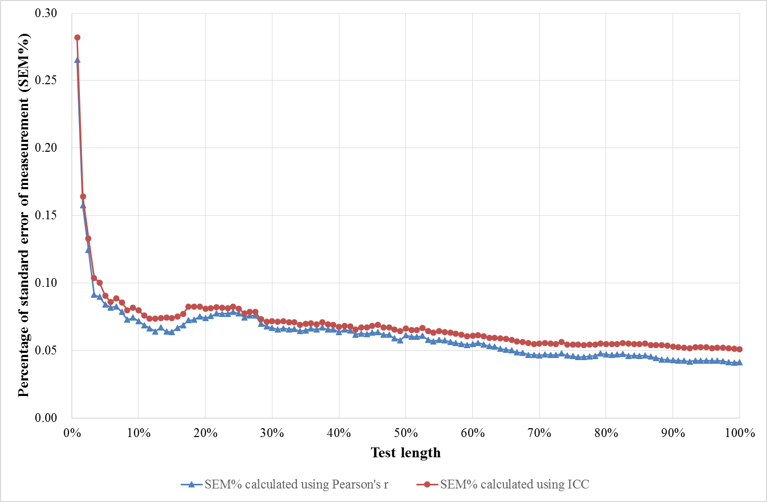

Supplement: S3 Appendix — The SEM% of each dot is the cumulative reliability (calculated from the first trial to that trial). (TIF) [file pone.0192922.s003.tif]
